# Black Ginger (*Kaempferia parviflora*) Extract Enhances Endurance Capacity by Improving Energy Metabolism and Substrate Utilization in Mice

**DOI:** 10.3390/nu14183845

**Published:** 2022-09-17

**Authors:** Jiapeng Huang, Takashi Tagawa, Sihui Ma, Katsuhiko Suzuki

**Affiliations:** 1 Graduate School of Sport Sciences, Tokorozawa Campus, Waseda University, Tokorozawa 3591192, Japan; 2 Maruzen Pharmaceuticals Co., Ltd., Hiroshima 7293102, Japan; 3 Faculty of Sport Sciences, Tokorozawa Campus, Waseda University, Tokorozawa 3591192, Japan

**Keywords:** *Kaempferia parviflora*, exercise tolerance, energy metabolism, lipid metabolism, lactate acid

## Abstract

Black ginger (*Kaempferia parviflora*) extract (KPE), extracted from KP, a member of the ginger family that grows in Thailand, has a good promotion effect on cellular energy metabolism and therefore has been used to enhance exercise performance and treatment of obesity in previous studies. However, the effect of single-dose administration of KPE on endurance capacity has not been thoroughly studied, and whether the positive effect of KPE on cellular energy metabolism can have a positive effect on exercise capacity in a single dose is unknown. In the present study, we used a mouse model to study the effects of acute KPE administration 1 h before exercise on endurance capacity and the underlying mechanisms. The purpose of our study was to determine whether a single administration of KPE could affect endurance performance in mice and whether the effect was produced through a pro-cellular energy metabolic pathway. We found that a single administration of KPE (62.5 mg/kg·bodyweight) can significantly prolong the exercise time to exhaustion. By measuring the mRNA expression of *Hk2, Slc2a4 (Glut4), Mct1, Ldh, Cd36, Cpt1β, Cpt2, Lpl, Pnpla2 (Atgl), Aco, Acadm (Mcad), Hadh, Acacb (Acc2), Mlycd (Mcd), Pparg, Ppargc1a (Pgc-1α), Tfam, Gp, Gs, Pfkm, Pck1 (Pepck), G6pc (G6pase), Cs,* and *Pfkl* in skeletal muscle and liver, we found that acute high-concentration KPE administration significantly changed the soleus muscle gene expression levels (*p* < 0.05) related to lipid, lactate, and glycogen metabolism and mitochondrial function. In gastrocnemius muscle and liver, glycogen metabolism-related gene expression is significantly changed by a single-dose administration of KPE. These results suggest that KPE has the potential to improve endurance capacity by enhancing energy metabolism and substrate utilization in muscles and liver.

## 1. Introduction

Black ginger (*Kaempferia parviflora*, KP) is a member of the ginger family that grows naturally in Laos and Thailand. The rhizome of KP is widely used in traditional medicine to treat diseases and symptoms such as inflammation, ulcers, gout, colic, and abscesses [1]. The KP extract (KPE), which is extracted from the rhizome of KP, contains several polymethoxy flavonoids (PMFs) [2]. PMFs belong to flavonoids in natural polyphenols [3], and the PMFs rich in KPE have been reported for a variety of pharmacological activities, such as suppressing the ability of prostate hyperplasia [4] and anti-tumor [5,6] and hepatoprotective effects [7]. In addition, the cellular-metabolism-regulating activity of PMFs has been reported in recent years [2,8,9,10]. According to the reports, KPE and/or PMFs promote cell energy metabolism by improving glucose and lipid metabolism and stimulating mitochondrial biogenesis [10,11,12,13]. KPE enhanced glycogen synthase mRNA expression and the content of glycogen in C2C12 cells, and in mice soleus muscle it also had a tendency to increase the content of glycogen [11]. It can inhibit fat accumulation in the obesity and diabetes model by inhibiting fat cell hypertrophy and activating thermogenesis function in brown adipose tissue [8,9,14,15,16]. KPE can also enhance the activation of AMP-activated protein kinase (AMPK) and phosphatidylinositol 3 kinase (PI3K)/Akt pathways to regulate muscle protein synthesis, and significantly increase the size, volume, and quality of muscle fibers, thereby enhancing muscle functions such as exercise endurance and grip strength [12,17,18].

Health-related components of physical fitness can be categorized as endurance, flexibility, balance, agility, and coordination, and endurance is standardized as cardiorespiratory endurance, muscle endurance, and muscle strength [19]. Great efforts are made by athletes and trainers to earn better endurance capacity, while endurance capacity is also important to endurance athletes as well very important to common individuals. Therefore, improvement of the overall endurance will also benefit the physical fitness of common people.

All movements of the human body require energy. Cells use material energy through adenosine triphosphate (ATP), which is called energy currency [20]. The continuous supply of ATP to the processes that support skeletal muscle contraction during exercise is critical to exercise performance lasting seconds to hours [21,22]. Intensity and duration of exercise are major determinants affecting the relative contributions of energy production pathways during exercise [21,22]. At lower exercise intensity, lipid oxidation dominates the energy supply, and the oxidation rate reaches a maximum when the exercise intensity is approximately 60–65% of VO_2_ max [22]. However, as the exercise intensity further increases to 80–100% VO_2_ max, carbohydrates take the lead of energy supply [21].

In long-term endurance exercise, the oxidative metabolism of carbohydrates and lipids provides almost all the adenosine triphosphate (ATP) that the body needs [22]. Carbohydrates used in endurance exercise are mainly produced by the decomposition of muscle glycogen, glucose, liver glycogen, and the cycle of gluconeogenesis [22]. The substrates for lipid oxidation are mainly derived from fatty acids from the breakdown of triglycerides (TG) in adipocytes and intramuscular triglycerides (IMTG) [23]. Lactate is regarded as an important substrate for oxidation and gluconeogenesis and plays an important role between glycolysis and oxidation [22]. In moderate- to high-intensity (70–75% VO_2_ max) prolonged exercise, blood lactate oxidation (90% direct oxidation, 10% through gluconeogenesis) accounts for about 30% of total carbohydrate metabolism [24,25]. Therefore, the availability and utilization of carbohydrate, lipid, and lactate in muscles and tissues are essential for maintaining athletic performance in endurance exercise.

Intake of nutritional supplements is one of the effective ways to enhance endurance capacity [26]. In recent years, the effects of various plant extracts on endurance capacity have been extensively studied. In several studies of plant extracts, the extracts of amarkand tubers, okra, and *tabebuia avellanedae* enhanced endurance performance in mice or rats by increasing hepatic or/and muscle glycogen storage and promoting antioxidant capacity [27,28,29].

Polyphenols, a large class of nutritional supplements, are ubiquitously produced in plants. The ability of polyphenols to upregulate exogenous antioxidants, enhance vascular function, and reduce oxidative damage during exercise [30] is believed to have potential benefits for exercise performance [31]. The polyphenolic compound PMF in KPE has also been reported to have potential benefits to exercise performance by enhancing mitochondria biogenesis, energy metabolism, or anti-inflammation [10,17,32,33]. However, all the subjects in the above experiment received long-term administration of KPE for at least one month. The effect of a single KPE administration on exercise performance has not been fully studied and whether the cellular energy metabolism regulation of KPE can be effective in a single administration remains to be verified. In a pharmacokinetic study of the main components of KPE, the concentration of KPE quickly reached a peak and was widely distributed in various organs within 1 to 2 h after oral administration and then slowly eliminated in rats [34]. Therefore, it is reasonable that KPE affects endurance performance by exerting its cellular metabolism regulation effects in 1–2 h after acute administration. It is necessary to study the effect of a single dose of KPE on the endurance of mice. The purpose of our study was to determine whether a single administration of KPE could affect endurance performance in mice and whether the effect was produced through a pro-cellular energy metabolic pathway. We designed this experiment and assumed that mice extend their exhaustive exercise time after a single KPE administration and evaluated the effect of KPE from the perspective of energy metabolism.

## 2. Materials and Methods

### 2.1. Animals

Male C57BL/6J mice (8 weeks old) were purchased from Takasugi experimental animals supply (Kasukabe, Japan) and were allowed to adapt to the environment for a week before the start of the formal experiment. Four or five animals were housed together in a cage (27 × 17 × 13 cm) in a controlled environment under a light–dark cycle (12 h of light (08:00–20:00)). The experimental procedure followed the Guiding Principles of Animal Care and Use of the Academic Research Ethics Review Committee of Waseda University and was approved (2020-A29).

### 2.2. Kaempferia parviflora Preparation

*Kaempferia parviflora* extract (KPE) was provided by Maruzen Pharmaceuticals Co, LTD (Hiroshima, Japan). Through strict process control in the production process and complete analysis control of the obtained dry extract, the standardization and compliance of the extract of Kaempferia parviflora were ensured. The γ-Cyclodextrin administration (γ-CD) was used as a control because administering γ-CD was necessary to uniformly disperse KPE in the solvent.

Supplement constituents:

γ-Cyclodextrin administration (γ-CD): 390 mg/kg·BW γ-CD;

Low-concentration KPE administration (LKPE): 387.5 mg/kg·BW (≒390 mg) γ-CD + 12.5 mg/kg·BW KPE (content 10 mg/kg·BW PMFs);

High-concentration KPE administration (HKPE): 387.5 mg/kg·BW (≒390 mg) γ-CD + 62.5 mg/kg·BW KPE (content 50 mg/kg·BW PMFs).

### 2.3. Experimental Protocol

All the mice were accustomed to a motorized treadmill (Kyoto, Japan) by running at 15 m/min for 10 min one week before the exhaustive exercise. Before the exercise experiment, all the animals were given normal feed.

On the day of the experiment, all mice were randomly divided into one of the three groups: KPE control (γ-CD) group (Con), LKPE group, and HKPE group. Mice in each group were then randomly divided into sedentary and exercise groups. Con group was divided into C group (γ-Cyclodextrin administration + sedentary group, *n* = 8) and E group (γ-Cyclodextrin administration + exercise group, *n* = 8). LKPE group was divided into L group (low-concentration KPE administration + sedentary group, *n* = 8) and LE group (low-concentration KPE administration + exercise group, *n* = 8). HKPE group was divided into H group (high-concentration KPE administration + sedentary group, *n* = 8) and HE group (high-concentration KPE administration + exercise group, *n* = 8). The sample size (*n* = 8) was determined by a preliminary test and our previous studies [35,36]. One hour before the exhaustive exercise, supplement was dissolved in distilled water at room temperature and was given by oral administration (dosage = BW×5μL) based on body weight (C: 24.775 ± 1.242 g; E: 24.7375 ± 1.238 g; L: 24.575 ± 1.103 g; LE: 25.1125 ± 1.249 g; H: 24.857 ± 1.338 g; HE: 24.6875 ± 1.119 g). C and E groups were given γ-CD (KPE compound as control), L and LE groups were given LKPE, and H and HE groups were given HKPE. Then, mice in the E, LE, and HE groups were subjected to treadmill running at 7% grade 10 m/min for 15 min, followed by 15 min at 15 m/min and then 20 m/min, followed finally by running at 24 m/min until exhaustion.

Exhaustion was defined as the inability to continue regular treadmill running despite the stimulation of repeated tapping on the back of the mouse. The time of exhaustive running was recorded. Mice were dissected under light anesthesia with the inhalant isoflurane (Abbott, Tokyo, Japan) immediately after the exhaustive treadmill running. Blood sample was taken using heparin from the abdominal aorta, and liver, soleus muscle, and gastrocnemius muscle were immediately excised and frozen in liquid nitrogen. Plasma was obtained from blood samples by centrifuging at 1500× *g* for 10 min at 4 °C. All the samples were stored at −80 °C until analyses.

### 2.4. Measurement of Plasma Biochemical Parameters, Liver Glycogen, and Biomarker of Oxidative Stress

Plasma concentration of triglyceride (TG), glucose, total cholesterol (TC), low-density lipoprotein cholesterol (LDL), high-density lipoprotein cholesterol (HDL), uric acid, blood urea nitrogen (BUN), creatinine (Cr), aspartate transaminase (AST), alanine transaminase (ALT), albumin, lactate dehydrogenase (LDH), creatine kinase (CK), amylase, and lipase were measured by Kotobiken Medical Laboratories (Tokyo, Japan). Liver glycogen was measured by Glycogen Colorimetric/Fluorometric Assay Kit (BioVision, Inc, Milpitas, CA, USA). The marker of oxidative stress in plasma was assessed using thiobarbituric acid reactive substances (Cayman Chemical, Ann Arbor, MI, USA).

### 2.5. Real-Time Quantitative Polymerase Chain Reaction (PCR)

Total RNA was extracted from the liver using RNeasy Mini Kit (Qiagen, Valencia, CA, USA), and gastrocnemius muscle and soleus muscle using the RNeasy Fibrous Mini Kit (Qiagen, Valencia, CA, USA) according to the manufacturer’s instructions. The concentration and purity of total RNA were assessed using the NanoDrop system (NanoDrop Technologies, Wilmington, DE, USA). Total RNA was reverse transcribed to cDNA using the High-Capacity cDNA Reverse Transcription Kit (Applied Biosystems, Foster City, CA, USA) according to the manufacturer’s instructions. The polymerase chain reaction (PCR) was performed with the Fast 7500 real-time PCR system (Applied Biosystems, Foster, CA, USA) using the Fast SYBR^®^ Green PCR Master Mix (Applied Biosystems, Foster, CA, USA). The thermal profiles consisted of 10 min at 95 °C for denaturation followed by 40 cycles of 95 °C for 3 s and annealing at 60 °C for 15 s; 18 s mRNA was used as the housekeeping gene, and the ΔΔCT method was used to quantify target gene expression. All data were represented relative to its expression as fold change based on the values of the C group.

PCR primer pairs for each studied gene are shown in Table 1.

### 2.6. Statistical Analysis

Data are presented as means ± standard deviation (SD). Independent samples test was performed to determine the main effects of KPE administration on exercise time. A two-way analysis of variance (ANOVA) was performed to determine the main effects of KPE administration and/or exercise. Statistical analysis was conducted using GraphPad Prism 9.0 (GraphPad, Ltd., LaJolla, CA, USA). When this analysis revealed significant interaction, simple effects analysis and Tukey’s post hoc test were performed to determine the significance of the means. When this analysis did not reveal a significant interaction, main effect analysis and Tukey’s post hoc test were performed to determine the significance of the means. Statistical significance was defined as *p* < 0.05.

## 3. Results

### 3.1. Effect of KPE Administration on Mice Endurance Capacity

**Figure 1:**
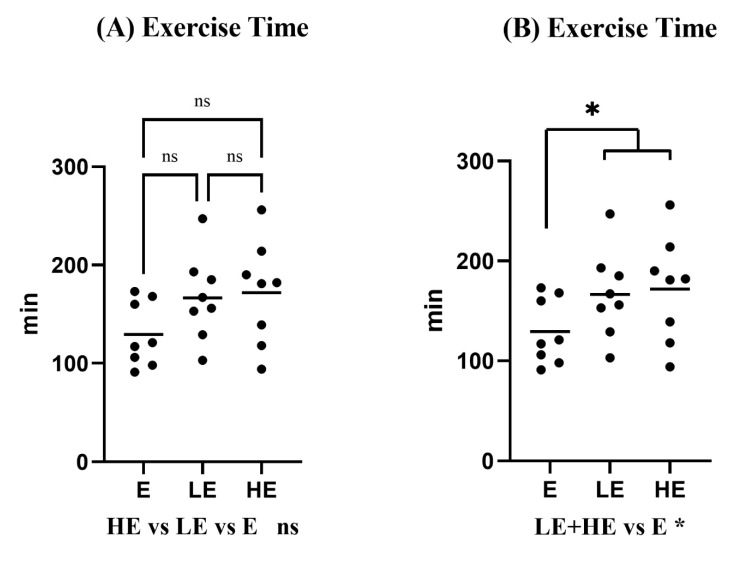
(**A**). The run time to exhaustion in between-group comparison. (**B**). The run time to exhaustion between the E group (*n* = 8) and KPE administration groups (LE group (*n* = 8) + HE group (*n* = 8)). E group, γ-Cyclodextrin administration + exercise group; LE group, low-concentration KPE administration + exercise group; HE group, high-concentration KPE administration + exercise group. Values are means ± standard deviation (SD); ns, no significance observed; * *p* < 0.05, compared with the E group; dots: the specific time each mice ran to exhaustion.

No significant difference was found between the concentration of KPE and the running time in the comparison among the HE, LE, and E groups (Figure 1A: 172 ± 53 min vs. 167 ± 44 min vs. 129 ± 33 min, *p >* 0.05). However, the running time to exhaustion of mice was significantly longer in the KPE administration group (HE + LE) than the E group (Figure 1B: 172 ± 53 min, 167 ± 44 min vs. 129 ± 33 min, *p* = 0.042). A single dose of HKPE administration enhanced mice endurance capacity by extending 33% of running time.

### 3.2. Effect of Exhaustive Exercise and KPE Administration on Metabolism Regulation in Plasma

**Figure 2:**
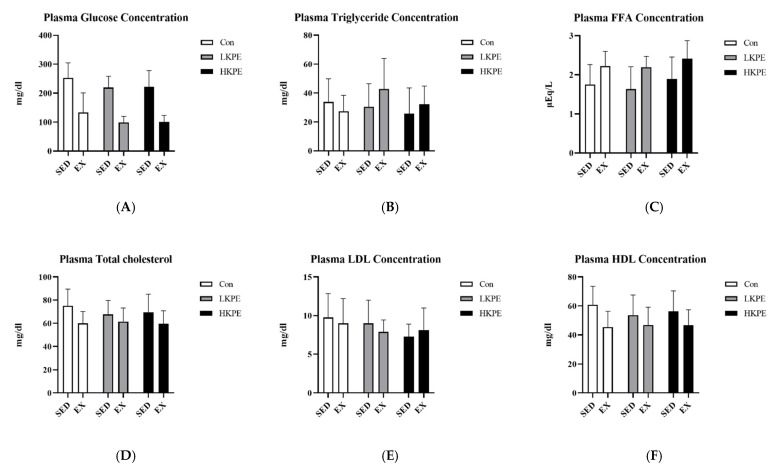
(**A**) Plasma glucose; (**B**) plasma triglyceride; (**C**) plasma FFA; (**D**) plasma total cholesterol; (**E**) plasma HDL; and (**F**) plasma LDL: concentrations immediately after exhaustion. FFA, free fatty acids; LDL, low-density lipoprotein cholesterol; HDL, high-density lipoprotein cholesterol; SED, sedentary; EX, exercise; Con, control; KPE, *Kaempferia parviflora* administration.

Exercise significantly increased plasma free fatty acids (FFA) (*p* < 0.001), while significantly decreased glucose (*p* < 0.001), total cholesterol (*p* < 0.01), and HDL (*p* < 0.01) in the plasma. Nevertheless, there were no significant changes with the administration of KPE (Figure 2).

### 3.3. Effect of Exhaustive Exercise and KPE Administration on Fatty-Acid-Metabolism-Related Gene Expression in Soleus Muscle

**Figure 3:**
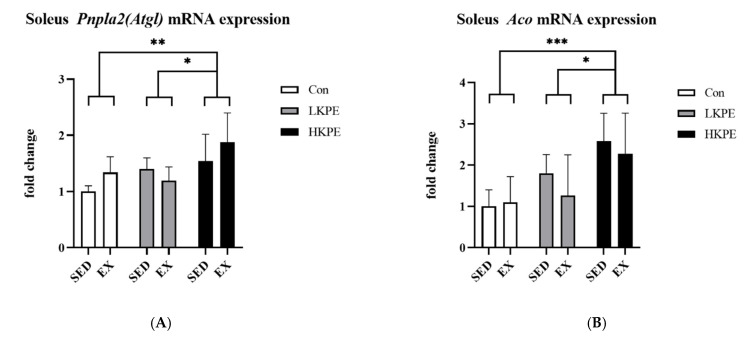

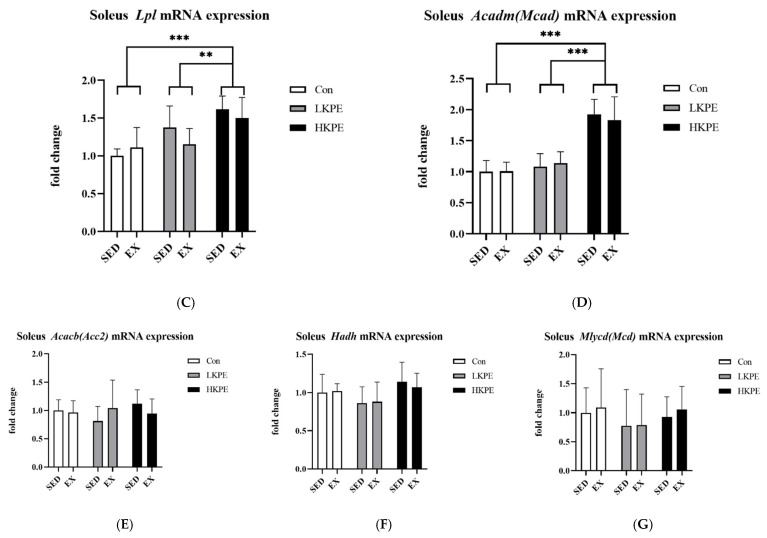
mRNA expression of (**A**) *Pnpla2* *(Atgl)*, (**B**) *Aco*, (**C**) *Lpl*, (**D**) *Acadm* *(Mcad)*, (**E**) *Acacb (Acc2)*, (**F**) *Hadh*, and (**G**) *Mlycd* *(Mcd)* in soleus muscle immediately after exhaustion. *Lpl*, lipoprotein lipase; *Pnpla2 (Atgl)*, patatin-like phospholipase domain containing 2; *Aco*, acyl-CoA oxidase; *Acadm (Mcad)*, acyl-Coenzyme A dehydrogenase medium chain; *Hadh*, hydroxy acyl-CoA dehydrogenase; *Acacb (Acc2)*, acetyl-Coenzyme A carboxylase beta; *Mlycd (Mcd)*, malonyl-CoA decarboxylase; SED, sedentary; EX, exercise; Con, control; KPE, *Kaempferia parviflora* administration; * *p* < 0.05, ** *p* < 0.01, *** *p* < 0.001.

HKPE administration significantly increased the gene expression of *Lpl* (*p* < 0.001), *Pnpla2 (Atgl)* (*p* < 0.01), *Aco* (*p* < 0.001), and *Acadm (Mcad)* (*p* < 0.001). The gene expression level of *Hadh, Acacb (Acc2),* and *Mlycd (Mcd)* did not show significant changes by KPE administration or exercise (Figure 3).

### 3.4. Effect of Exhaustive Exercise and KPE Administration on Fatty Acid Transmembrane Transport Related Gene Expression in Soleus Muscle

**Figure 4:**
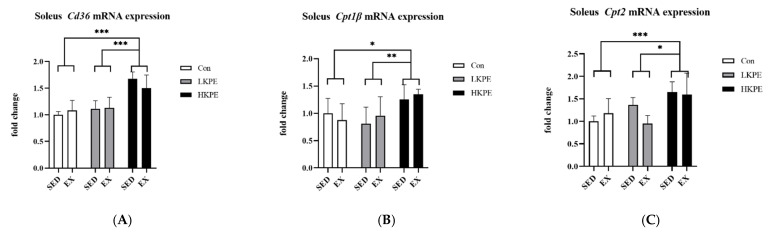
mRNA expression of (**A**) *Cd36*, (**B**) *Cpt1*β, and (**C**) *Cpt2* in soleus muscle immediately after exhaustion. *Cd36*, CD36 molecule; *Cpt*, carnitine palmitoyl transferase; SED, sedentary; EX, exercise; Con, control; KPE, *Kaempferia parviflora* administration; * *p* < 0.05, ** *p* < 0.01, *** *p* < 0.001.

Compared to other groups, HKPE administration groups showed significant higher gene expression levels of *Cd36* (*p* < 0.001), *Cpt1β (p* < 0.01), and *Cpt2 (p* < 0.001) (Figure 4).

### 3.5. Effect of Exhaustive Exercise and KPE Administration on Glucose-Metabolism-Related Gene Expression in Soleus Muscle

**Figure 5:**
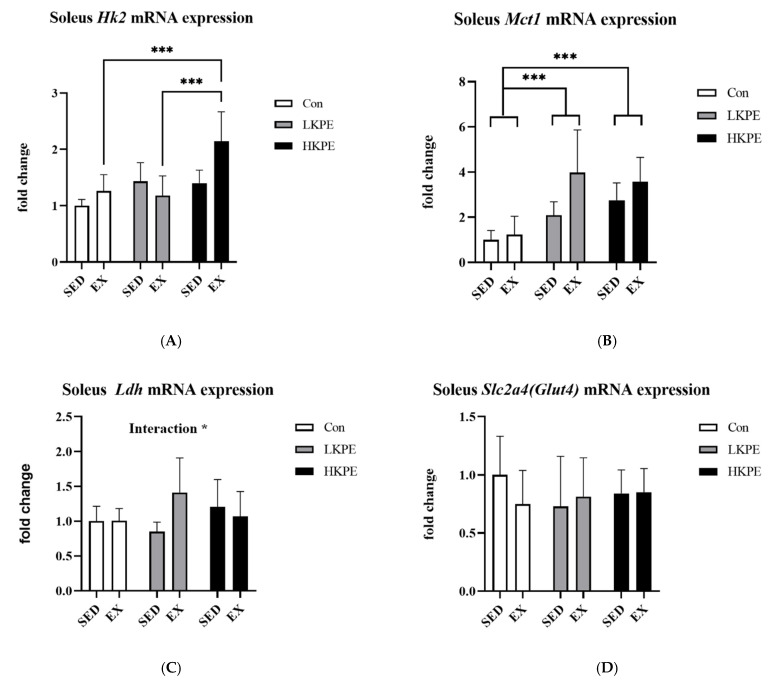
mRNA expression of (**A**) *Hk2*, (**B**) m *Mct1*, (**C**) *Ldh,* and (**D**) *Slc2a4* *(Glut4)* in soleus muscle immediately after exhaustion. *Hk2*, hexokinase 2; *Slc2a4 (Glut4)*, solute carrier family 2 (facilitated glucose transporter); *Mct1*, monocarboxylate transporter 1; *Ldh*, lactate dehydrogenase; SED, sedentary; EX, exercise; Con, control; KPE, *Kaempferia parviflora* administration; * *p* < 0.05, *** *p* < 0.001.

The hexokinase 2 (*Hk2*) gene expression level in the HE group was significantly higher than that in E or LE groups (*p* < 0.001). Exercise also increased the *Hk2* gene expression level (*p* < 0.05). The mRNA expression of *Mct1* was significantly increased after exercise (*p* < 0.01) and KPE administration (*p* < 0.001). The interaction between exercise and of KPE administration significantly increased *Ldh* gene expression (*p* < 0.05). There was no significant change in *Slc2a4* *(Glut4)* gene expression by exercise or KPE administration (Figure 5).

### 3.6. Effect of Exhaustive Exercise and KPE Administration on Mitochondrial-Function-Related Gene Expression in Soleus Muscle

**Figure 6:**
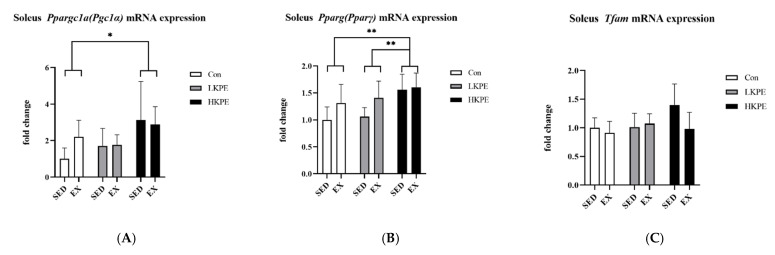
mRNA expression of (**A**) *Ppargc1a (Pgc-1α)*, (**B**) *Pparγ*, and (**C**) *Tfam* in soleus muscle immediately after exhaustion. *Pparγ*, peroxisome proliferator-activated receptor gamma; *Ppargc1a (Pgc-1α)*, peroxisome proliferator-activated receptor gamma coactivator 1-alpha; *Tfam*, mitochondrial transcription factor A; SED, sedentary; EX, exercise; Con, control; KPE, *Kaempferia parviflora* administration; * *p* < 0.05, ** *p* < 0.01.

The gene expression of *Pparγ* was significantly increased by HKPE administration (*p* < 0.01) and exercise (*p* < 0.05). The *Ppargc1a (Pgc-1α)* gene expression was also significantly enhanced by HKPE administration (*p* < 0.05). Exercise or KPE administration did not have a significant effect on the *Tfam* gene expression (Figure 6).

### 3.7. Effect of Exhaustive Exercise and KPE Administration on Glucose-Metabolism-Related Gene Expression in Gastrocnemius Muscle

**Figure 7:**
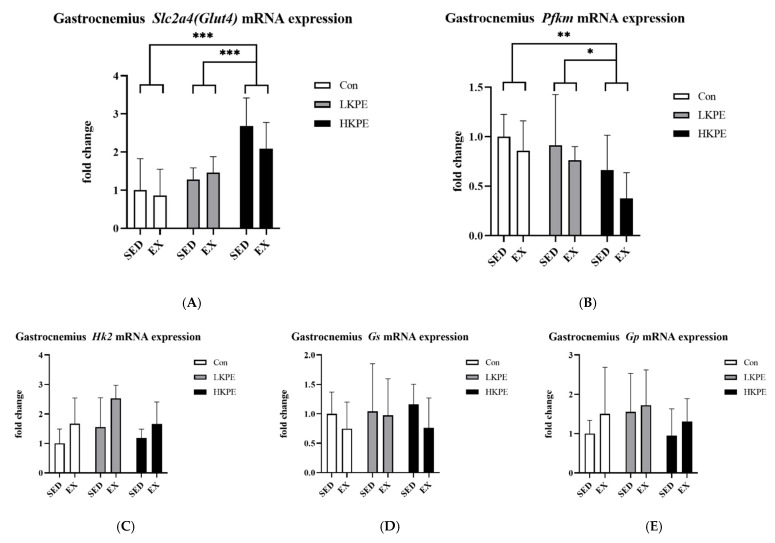
mRNA expression of (**A**) *Slc2a4* *(Glut4)*, (**B**) *Pfkm*, (**C**) *Hk2*, (**D**) *Gs*, and (**E**) *Gp* in gastrocnemius muscle immediately after exhaustion. *Gs*, glycogen synthase; *Gp*, glycogen phosphorylase; *Pfkm*, 6-phosphofructokinase muscle type; *Hk2*, hexokinase 2; *Slc2a4 (Glut4),* solute carrier family 2 (facilitated glucose transporter); SED, sedentary; EX, exercise; Con, control; KPE, *Kaempferia parviflora* administration; * *p*< 0.05, ** *p*< 0.01, *** *p* < 0.001.

HKPE groups had significantly increased *Slc2a4* *(Glut4)* (*p* < 0.001) and decreased *Pfkm* (*p* < 0.01) gene expression levels. Exercise increased *Hk2* gene expression (*p* < 0.01). Exercise or KPE administration did not have a significant effect on the *Gp* and *Gs* gene expression (Figure 7).

### 3.8. Effect of Exhaustive Exercise and KPE Administration on Glycogen-Metabolism-Related Gene Expression in Liver

**Figure 8:**
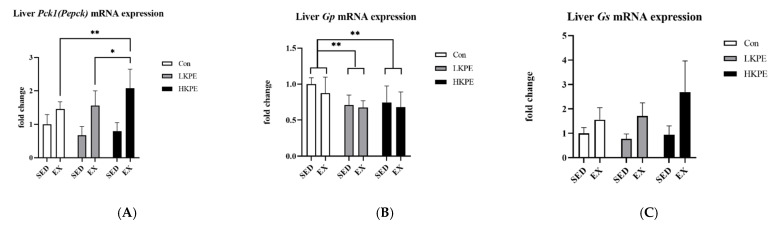

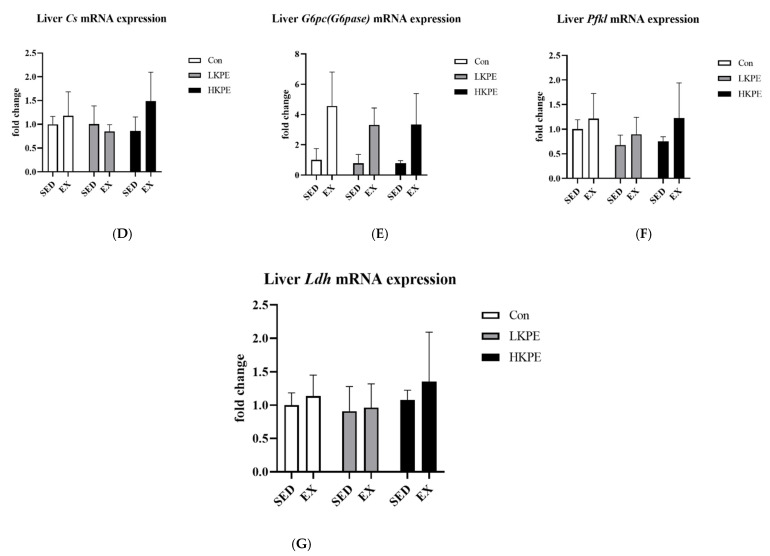
mRNA expression of (**A**) *Pck1* *(Pepck)*, (**B**) *Gp*, (**C**) *Gs*, (**D**) *Cs*, (**E**) *G6pc (G6pase)*, (**F**) *Pfkl*, and (**G**) *Ldh* in liver immediately after exhaustion. *G6pc (G6pase)*, glucose-6-phosphatase catalytic; *Pck1 (Pepck),* phosphoenolpyruvate carboxykinase 1 cytosolic; *Gs*, glycogen synthase; *Gp*, glycogen phosphorylase; *Pfkl*, 6-phosphofructokinase liver type; *Gs*, citrate synthase; *Ldh*, lactate dehydrogenase; SED, sedentary; EX, exercise; Con, control; KPE, Kaempferia parviflora administration; * *p* < 0.05, ** *p* < 0.01.

HKPE administration significantly increased the hepatic *Gs* (*p* < 0.05) and decreased *Gp* (*p* < 0.01) gene expression level. The interaction between exercise and HKPE administration significantly increased hepatic *Pck1 (Pepck)* gene expression (*p* < 0.05). Exercise also significantly increased hepatic *G6pc* *(G6pase)*, *Gs*, and *Pfkl* gene expression (Figure 8).

## 4. Discussion

This study describes the effect of a single administration of different concentrations of KPE on the endurance of mice one hour before exercise and the possible mechanisms. Our study found that a single dose of KPE administration enhanced endurance capacity in mice (Figure 1). Although there was no difference in exercise time between different concentrations of KPE administration, significant differences in gene expression compared with the control group were mainly high-concentration groups (C vs. HKPE). This may indicate that compared with LKPE, HKPE significantly affected the energy metabolism pathways of mice.

Regarding plasma biochemical data (Figure 2), exhaustive exercise seems to significantly induce damage in various organs, but KPE does not seem to have an immediate effect on anti-inflammatory or antioxidant properties.

### 4.1. KPE Has the Potential to Improve Lipid and Lactate Transportation, Oxidation Capacity, Glycolysis and Mitochondrial Function in Soleus Muscle

Our study measured the soleus muscle because of its higher type 1 muscle fiber content [37], and type 1 muscle fiber displays higher fatigue resistance and lipid oxidative metabolism ability [38].

IMTG in muscle fibers and plasma FFA are the main metabolic fuels used for the contraction of skeletal muscle during endurance exercise [22]. Studies have shown that in trained individuals during long-term moderate-intensity exercise, type I muscle fibers consume 60–70% of IMTG, and the energy produced can account for 50% of total lipid oxidation [39]; the other half of the lipid consumption basically comes from FFAs [40]. Therefore, for the soleus muscle, which has a high proportion of type I muscle fibers [38], the energy source during exercise is inseparable from the utilization of IMTG and FFAs.

Adipose triglyceride lipase (ATGL) is the key enzyme that regulate lipolysis [39], while lipoprotein lipase (LPL) mediates intravascular hydrolysis of triglycerides packed in lipoproteins such as chylomicrons and very-low-density lipoprotein (VLDL) [41]. In our study, the administration of KPE significantly increased the gene expression of *Pnpla2* *(Atgl)* and *Lpl* (Figure 3A,C), partly consistent with the conclusions of Okabe et al. [15] in adipocytes in a previous study. These data indicate that KPE has the potential to promote the initial decomposition of various lipids (FFAs, IMTG, VLDL, etc.) in the soleus muscle.

Fatty acids (LCFA) produced by lipolysis outside the cell need to be transported across the cell membrane by the CD36 molecule (CD36) [42] to enter the cell, and the transport of LCFA-CoA across the mitochondrial membrane is based on the CPT system [22]. The CPT1β and CPT2 enzyme transports LCFA across the mitochondrial outer and inner membrane, respectively [43]. Then, further fatty acid β-oxidation by *Mcad* and *Aco* enzymes [44,45] is required in mitochondria to convert LCFA-CoA into acetyl-CoA to enter the tricarboxylic acid (TCA) cycle [46].

Our study found that KPE supplementation remarkably increased the mRNA expression of *Cd36, Cpt1β, Cpt2*, *Acadm (Mcad)*, and *Aco* in the mice soleus muscle (Figure 3B,D and Figure 4A–C). It was demonstrated that KPE had the potential to enhance LCFA transmembrane transportation capacity and lipid oxidation.

Lactate accumulated in muscles during exercise, which can be removed by the catalysis of LDH [47] or via the pH-dependent monocarboxylate transport (MCT) system to excrete lactate into circulation [48]. In addition, the lactate intake from the circulation is also highly correlated with the MCT1 content of muscles (especially slow-twitch fibers, such as the soleus muscle) [49]. HK2 is an important rate-limiting enzyme catalyzing glucose metabolism and glycolysis, a subtype of hexokinase (HKs), widely distributed in cardiac and skeletal muscle [50].

In our study, KPE supplementation increased *Mct1* and HK2 mRNA expression level in the soleus muscle (Figure 5A–C), which was consistent with some results of the previous study [11], and the interaction with exercise enhances the *Ldh* level of mRNA. These data suggested that ingestion of KPE has the potential to improve the lactate metabolism, transport capacity, and glycolysis of the soleus muscle of mice.

KPE has been proven to enhance exercise performance through activation of mitochondrial biogenesis [10], while PGC-1α and PPARγ are among the major regulators [51]. PPARγ is also the main regulator of systemic lipid metabolism, adipogenesis, and insulin sensitivity, and the expression of CD36 is enhanced by the activation of PPARγ [42]. PGC-1α in skeletal muscle increases skeletal muscle lactate uptake by increasing MCT1 expression [48] and controls systemic lactate levels during maximal endurance performance testing [52].

In our study, the administration of KPE significantly increased the mRNA expression of *Pparγ* and *Ppargc1a (Pgc-1α)* in the soleus muscle of mice (Figure 6A,B), partly consistent with the conclusions of previous studies [10]. It indicated that KPE may affect the expression of *Cd36* and *Mct1* genes by upregulating the expression of these two genes, thereby promoting lactate uptake and lipid metabolism by altering mitochondrial function.

**Figure 9:**
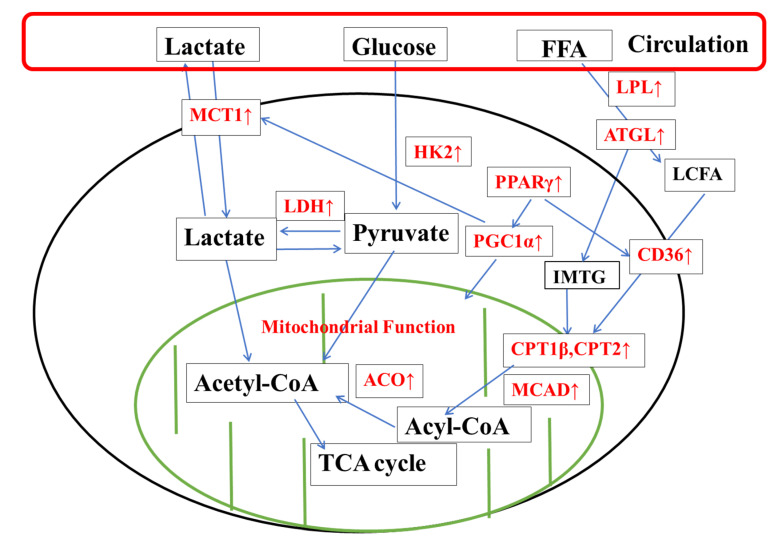
The suggested mechanisms by KPE administration enhance cell metabolism in soleus muscle. FFA, free fatty acids; LPL, lipoprotein lipase; ATGL, Adipose triglyceride lipase; MCT1, monocarboxylate transporter 1; Hk2, hexokinase 2; LCFA, Fatty acids; PPARγ, peroxisome proliferator-activated receptor gamma; LDH, lactate dehydrogenase; PGC1α, peroxisome proliferator-activated receptor gamma coactivator 1-alpha; Cd36, CD36 molecule; IMTG, intramuscular triglycerides; CPT, carnitine palmitoyl transferase; Acetyl-CoA, acetyl coenzyme A; ACO, acyl-CoA oxidase; MCAD, acyl-Coenzyme A dehydrogenase medium chain; TCA cycle, tricarboxylic acid cycle. Arrows, significantly altered gene expression due to KPE administration.

In conclusion, in the soleus muscle, the mechanism of action of KPE may be through upregulating the levels of genes related to energy metabolism, improving mitochondrial function and utilization of lipids, lactate, and carbohydrates, which ultimately manifests itself as increasing the time from exercise to exhaustion (Figure 9).

### 4.2. KPE Has the Potential to Regulate Glucose Transport in Gastrocnemius Muscle and Liver

The major fibers of gastrocnemius muscle are IIa, IIb, and IIx fibers with fast contraction rates and high enzymatic activity of the glycolytic system [53], which play a greater role in the final phase of exhaustive exercise [37]. According to prior research [12], our study focused on measuring the expression of genes related to glucose metabolism in the gastrocnemius muscle.

The final phase of exhaustive exercise is associated with a high rate of glycolysis, which lowers pH through the accumulation of [H^+^] and affects the stability of muscle metabolism [54]. *Pfkm* is a key regulatory enzyme in muscles responsible for promoting glycolysis [55,56] of muscle glycogen and blood glucose transported into cells by the glucose transporter GLUT4 [57,58]. In our study, we found that HKPE administration significantly increased the *Slc2a4 (Glut4)* but decreased *Pfkm* mRNA expression(Figure 7A,B). KPE may promote glucose transport and inhibit glycolysis in the gastrocnemius muscle by upregulating the expression of these genes to improve gastrocnemius muscle fatigue resistance by delaying the rise in glycolytic flux during exercise [54,59].

The liver is the body’s largest glycogen reservoir and the tissue with the greatest capacity for gluconeogenesis [60]. During exercise, the liver maintains blood glucose through glycogenolysis and gluconeogenesis and provides glucose to peripheral tissues [61]. Therefore, in the liver, our study mainly measured the gene expression levels of factors related to gluconeogenesis, glycogenolysis, and synthesis.

Our study found that KPE significantly increased the gene expression of *Gs* and decreased *Gp* (Figure 8B,C). Because *Gp* and *Gs* catalyze the phosphorolysis reaction and synthesis of glycogen, respectively [29], KPE administration may promote glycogen storage in the liver. PEPCK is the rate-limiting enzyme of gluconeogenesis and maintains glucose homeostasis by catalyzing glucose synthesis, and the activity of the TCA cycle is directly related to the abundance of PEPCK [62]. Because the interaction between KPE and exercise significantly increased the gene expression of *Pck1 (Pepck)* (Figure 8A)*,* the rate of gluconeogenesis may be altered by its synergistic effect.

Our study speculates that KPE may interact with exercise and enhances the ability of liver gluconeogenesis and TCA circulation to make the lactate produced during exercise more effectively converted into glucose or glycogen in the liver [63]. In this way, the glucose homeostasis of the mice during exercise is maintained, so that the exercise time of the mice can be prolonged.

### 4.3. KPE Has the Potential to Improve Energy Metabolism and Substrate Utilization between Skeletal Muscle and Liver

Metabolites produced during exercise circulate between the working muscles and the liver and may support metabolism to adapt to exercise by acting as substrates and signaling molecules [64]. The metabolite lactate during exercise maintains an energy balance between tissues and muscles through glycolysis and aerobic metabolism [65].

The concept of lactate shuttling may help us to explain the role of HKPE in substrate utilization. HKPE promotes fast-twitch muscle fibers to convert more glucose to lactate by increasing glucose transport, and promotes the transportation, absorption, and utilization of lactate in slow-twitch fibers. It also promotes the gluconeogenesis of lactate in the liver, maintaining blood glucose, lactate levels, and muscle glucose requirements [65].

**Figure 10:**
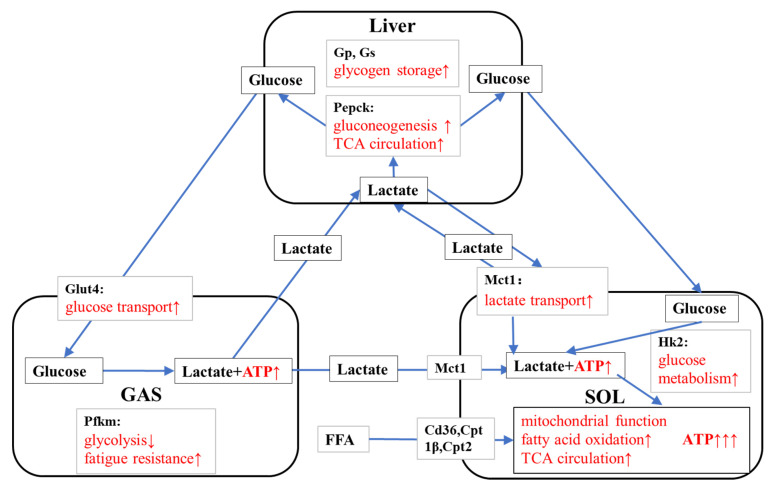
The suggested mechanisms of KPE administration enhancing energy metabolism and substrate utilization. Gs, glycogen synthase; GP, glycogen phosphorylase; Pepck, phosphoenolpyruvate carboxykinase 1 cytosolic; Pfkm, 6-phosphofructokinase muscle type; MCT1, monocarboxylate transporter 1; Glut4, solute carrier family 2 (facilitated glucose transporter); Hk2, hexokinase 2; Cd36, CD36 molecule; CPT, carnitine palmitoyl transferase; FFA, free fatty acids; GAS, gastrocnemius muscle; SOL, soleus muscle. Arrows, Potential effects of KPE administration.

In our study, the plausible mechanism of HKPE that prolongs the exercise-to-failure time in mice may be because HKPE promotes the balance of substrate utilization between liver and skeletal muscle by activating the expression of genes related to energy metabolism in liver and skeletal muscle (Figure 10). However, the metabolites in plasma did not change significantly due to the KPE administration. This is possibly due to the limited effect of a single dose in mice, which was not sufficient to induce changes in plasma metabolites.

The limitation of this study is that it only collected relevant data after the end of exercise instead of the changes in substrate turnover in mice during exercise, and further research on this is encouraged. Another limitation is that only a single administration was performed in this experiment, and the effect of long-term administration of KPE on energy metabolism in mice requires further study. Regarding statistical analysis, we followed previous studies using ANOVA, but according to the characteristics of the variables, we thought non-parametric statistics would have been the most appropriate. We were unable to measure metabolite levels in plasma and muscle due to lack of sufficient samples, which should be conducted in a follow-up study. We used only males in the present study, and further study is encouraged using female subjects. To further understand the underlying mechanisms, a comprehensive analysis using metabolomic techniques is favored.

## 5. Conclusions

In this study, a single dose of KPE administration enhanced the endurance ability of mice. HKPE showed more effects on the energy metabolism pathways of mice. The possible mechanism for increased endurance capacity may be that single-dose HKPE administration promotes glucose, lactate, and lipid metabolism and regulates mitochondrial function in liver and muscle to regulate energy metabolism and substrate utilization.

## Figures and Tables

**Table 1:** Primer sequence for Real-Time PCR analysis.

Gene Symbol for Primer	Forward	Reverse
*Hk2*	CTGTCTACAAGAAACATCCCCATTT	CACCGCCGTCACCATAGC
*Slc2a4 (Glut4)*	CCGCGGCCTCCTATGAGATACT	AGGCACCCCGAAGATGAGT
*Mct1*	GTGACCATTGTGGAATGCTG	CTCCGCTTTCTGTTCTTTGG
*Ldh*	ACAGTTGTTGGGTTGGTG	CCGGCTCTGCCCTCTTG
*Cd36*	TGGCCTTACTTGGGATTGG	CCAGTGTATATGTAGGCTCATCCA
*Cpt1β*	CCCATGTGCTCCTACCAGAT	CCTTGAAGAAGCGACCTTTG
*Cpt2*	GAAGAAGCTGAGCCCTGATG	GCCATGGTATTTGGAGCACT
*Lpl*	GAGAAGCCATCCGTGTGATT	TATGCTTTGCTGGGGTTTTC
*Pnpla2 (Atgl)*	GAGCCCCGGGGTGGAACAAGAT	AAAAGGTGGTGGGCAGGAGTAAGG
*Aco*	TGTTAAGAAGAGTGCCACCA	ATCCATCTCTTCATAACCAAATTT
*Acadm (Mcad)*	GCTCGTGAGCACATTGAAAA	CATTGTCCAAAAGCCAAACC
*Hadh*	ACTACATCAAAATGGGCTCTCAG	AGCAGAAATGGAATGCGGACC
*Acacb (Acc2)*	GGGCTCCCTGGATGACAAC	TTCCGGGAGGAGTTCTGGA
*Mlycd (Mcd)*	ACTCCATCAGCCTGACCCAG	ACCCCTTGAGGCTCTCGTGA
*Pparg*	GATGGAAGACCACTCGCATT	AACCATTGGGTCAGCTCTTG
*Ppargc1a* *(Pgc-1α)*	GACTGGAGGAAGACTAAACGGCCA	GCCAGTCACAGGAGGCATCTTT
*Tfam*	CCAAAAAGACCTCGTTCAGC	CTTCAGCCATCTGCTCTTCC
*Gp*	TGGCAGAAGTGGTGAACAATGAC	CCGTGGAGATCTGCTCCGATA
*Gs*	ACTGCTTGGGCGTTATCTCTGTG	ATGCCCGCTCCATGCGTA
*Pfkm*	GGAGTGCGTGCAGGTGACCAAA	ATCACGGCCACTGTGTGCAACC
*Pck1 (Pepck)*	CACCATCACCTCCTGGAAGA	GGGTGCAGAATCTCGAGTTG
*G6pc (G6pase)*	GTGGCAGTGGTCGGAGACT	ACGGGCGTTGTCCAAAC
*Cs*	GCATGAAGGGACTTGTGTA	TCTGGCACTCAGGGATACT
*Pfkl*	CATGAATGCAGCTGTGCGCTCC	CCAGCCCACTTCTTGCACCTGA
*18s*	CGGCTACCACATCCAAGGA	AGCTGGAATTACCGCGGC

*Hk2*, hexokinase 2; *Slc2a4(Glut4)*, solute carrier family 2 (facilitated glucose transporter), member 4; *Mct1*, monocarboxylate transporter 1; *Ldh*, lactate dehydrogenase; *Cd36*, CD36 molecule; *Cpt*, carnitine palmitoyl transferase; *Lpl*, lipoprotein lipase; *Pnpla2 (Atgl)*, patatin-like phospholipase domain containing 2; *Aco*, acyl-CoA oxidase; *Acadm (Mcad)*, acyl-Coenzyme A dehydrogenase medium chain; *Hadh*, hydroxyacyl-CoA dehydrogenase; *Acacb (Acc2)*, acetyl-Coenzyme A carboxylase beta; *Mlycd (Mcd),* malonyl-CoA decarboxylase; *Pparg*, peroxisome proliferator-activated receptor gamma; *Ppargc1a (Pgc-1α)*, peroxisome proliferator-activated receptor gamma coactivator 1-alpha; *Tfam,* mitochondrial transcription factor A; *Gs*, glycogen synthase; *GP*, glycogen phosphorylase; *G6pc (G6pase)*, glucose-6-phosphatase catalytic; *Pck1* *(Pepck)*, phosphoenolpyruvate carboxykinase 1 cytosolic; *Pfkm/l*, 6-phosphofructokinase muscle/liver type; *Cs*, citrate synthase; *18 s,* 18 s ribosomal RNA.

## Data Availability

Not applicable.
